# A human 3D immune competent full-thickness skin model mimicking dermal dendritic cell activation

**DOI:** 10.3389/fimmu.2023.1276151

**Published:** 2023-11-06

**Authors:** Johanna Maria Hölken, Katja Friedrich, Marion Merkel, Nelli Blasius, Ursula Engels, Timo Buhl, Karsten Rüdiger Mewes, Lars Vierkotten, Nicole Elisabeth Teusch

**Affiliations:** ^1^ Institute of Pharmaceutical Biology and Biotechnology, Heinrich Heine University Düsseldorf, Düsseldorf, Germany; ^2^ Alternative Methods and Tissue Engineering, Henkel AG & Co. KGaA, Düsseldorf, Germany; ^3^ Department of Dermatology, Venereology and Allergology, University Medical Center Göttingen, Göttingen, Germany

**Keywords:** full-thickness skin model, dermal dendritic cell, nickel, DNCB, CD86, p38 MAPK, NF-κB

## Abstract

We have integrated dermal dendritic cell surrogates originally generated from the cell line THP-1 as central mediators of the immune reaction in a human full-thickness skin model. Accordingly, sensitizer treatment of THP-1-derived CD14^-^, CD11c^+^ immature dendritic cells (iDCs) resulted in the phosphorylation of p38 MAPK in the presence of 1-chloro-2,4-dinitrobenzene (DNCB) (2.6-fold) as well as in degradation of the inhibitor protein kappa B alpha (IκBα) upon incubation with NiSO_4_ (1.6-fold). Furthermore, NiSO_4_ led to an increase in mRNA levels of IL-6 (2.4-fold), TNF-α (2-fold) and of IL-8 (15-fold). These results were confirmed on the protein level, with even stronger effects on cytokine release in the presence of NiSO_4_: Cytokine secretion was significantly increased for IL-8 (147-fold), IL-6 (11.8-fold) and IL-1β (28.8-fold). Notably, DNCB treatment revealed an increase for IL-8 (28.6-fold) and IL-1β (5.6-fold). Importantly, NiSO_4_ treatment of isolated iDCs as well as of iDCs integrated as dermal dendritic cell surrogates into our full-thickness skin model (SM) induced the upregulation of the adhesion molecule clusters of differentiation (CD)54 (iDCs: 1.2-fold; SM: 1.3-fold) and the co-stimulatory molecule and DC maturation marker CD86 (iDCs ~1.4-fold; SM:~1.5-fold) surface marker expression. Noteworthy, the expression of CD54 and CD86 could be suppressed by dexamethasone treatment on isolated iDCs (CD54: 1.3-fold; CD86: 2.1-fold) as well as on the tissue-integrated iDCs (CD54: 1.4-fold; CD86: 1.6-fold). In conclusion, we were able to integrate THP-1-derived iDCs as functional dermal dendritic cell surrogates allowing the qualitative identification of potential sensitizers on the one hand, and drug candidates that potentially suppress sensitization on the other hand in a 3D human skin model corresponding to the 3R principles (“replace”, “reduce” and “refine”).

## Introduction

Immune responses in the skin are mediated by antigen-presenting cells (APCs) such as macrophages, monocytes and most importantly dendritic cells (1). Cutaneous dendritic cells include epidermal Langerhans cells (LCs) and dermal dendritic cells (DDCs), located beneath the epidermal-dermal junction and throughout the dermis (2). While Langerhans cells are characterized by the expression of Langerin (3), to date, no specific marker exclusively expressed on all dermal dendritic subsets has been reported. However, dermal dendritic cells can be identified and distinguished from dermal monocytes and macrophages by a low CD14 expression and a high CD11c expression (4, 5). Yet, the primary and common function of all cutaneous dendritic cell subsets includes endocytosis/phagocytosis, processing and presenting antigens to naïve T cells (6).

**Figure 1:**
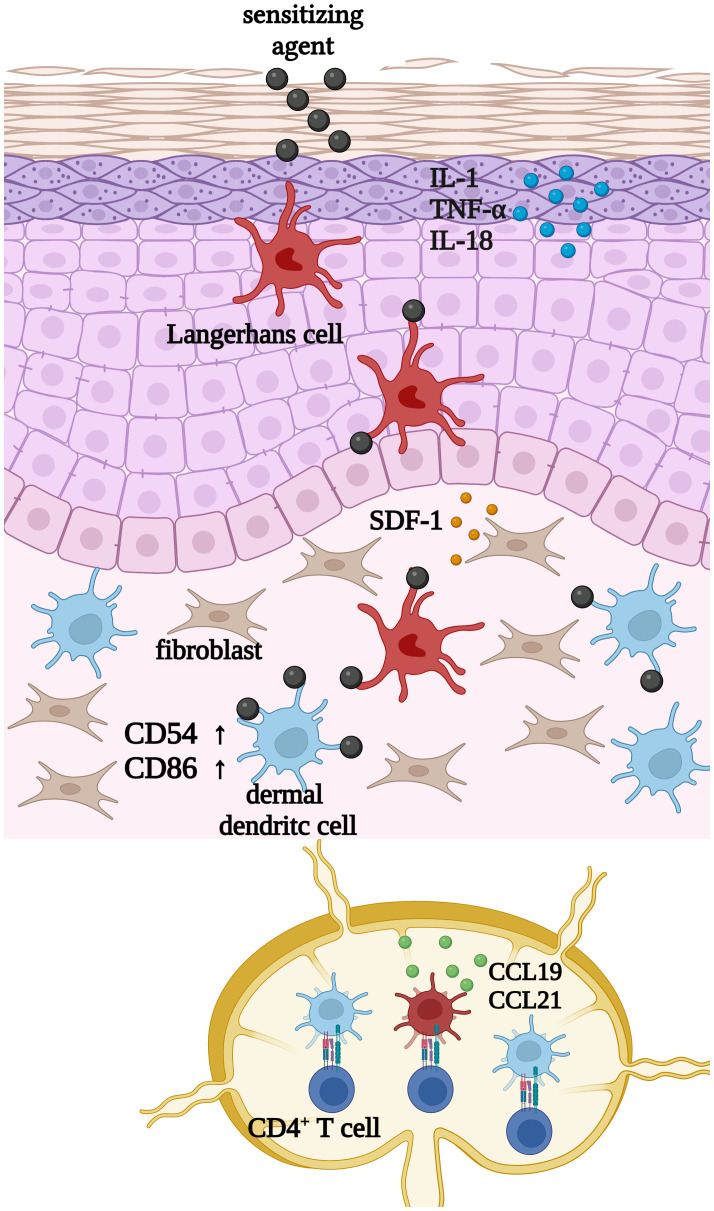
Upon exposure to inflammatory stimuli such as LPS, or sensitizing chemicals such as 1 chloro-2,4-dinitrobenzene (DNCB) or nickel sulfate (NiSO_4_), keratinocytes start to secrete inflammatory cytokines such as IL-1, TNF-α and IL-18. Subsequently cutaneous dendritic cells such as Langerhans cells and dermal dendritic cells become activated and start to phagocytose haptens or exogenous particles, which is accompanied by cell maturation and the upregulation of CD54 and CD86. Finally, DCs migrate to draining lymph nodes to present the processed antigen in order to activate CD4^+^ T cells. Created with BioRender.com.

The activation of cutaneous dendritic cells can be divided into different central events (**Figure 1**): Upon exposure to inflammatory stimuli like interleukin (IL)-1β, lipopolysaccharide (LPS) or sensitizing agents such as 1 chloro-2,4-dinitrobenzene (DNCB) or nickel sulfate (NiSO_4_), keratinocytes start to secrete a variety of cytokines including IL-1, TNF-α or IL-18 (7–9). Consequently, cutaneous dendritic cells such as Langerhans cells and dermal dendritic cells become activated and start to phagocytose the hapten accompanied by cell maturation and the upregulation of adhesion molecules, such as clusters of differentiation (CD)54 and co-stimulatory molecules including CD80 and CD86 (10, 11). Upregulation of the intracellular adhesion molecule (ICAM-1)/CD54 is required to form a stable signaling structure, the so-called immunological synapse (IS) with the leukocyte function-associated antigen 1 (LFA-1) alpha (CD11a) and beta-2 (CD18) in naïve CD4^+^ T cells (12). High expressions of CD80 (B7-1) and CD86 (B7-2) finally ensure the co-stimulation of naïve CD4^+^ T cells via their CD28 and cytotoxic T-lymphocyte-associated protein 4 (CTLA-4)/(CD152) receptors (13).

Regarding the underlying intracellular signal transduction, different studies with cord blood derived DCs as well as PBMC derived DCs were able to prove the central role of the mitogen-activated protein kinases (MAPK) pathway and the nuclear factor (NF)-κB pathway in skin sensitization and dendritic cell activation (14–16). Inhibition of the p38 MAPK pathway inhibited the upregulation of the DC maturation markers CD80, CD86 and CD83 in PBMC derived DCs (16). In addition, the up-regulation and secretion of IL-1, IL-8 and tumor necrosis factor (TNF)-α was suppressed (15, 16). Inhibition of the NF-κB pathway resulted in a downregulation of CD86 and CD83 and abolished the secretion of IL-8, IL-6 and IL-12p40 in cord blood derived DCs (14). While IL-8 functions as a chemotactic for neutrophils and T cells (17, 18), IL-6 is considered as a pleiotropic cytokine involved in DC maturation, T cell differentiation and proliferation as well as in B cell activation (19). TNF-α induces the expression of adhesion molecules such as vascular endothelial cell adhesion molecule (VCAM)-1 and ICAM-1 as well as T cell infiltration into the skin (20). IL-1 induces the expression of adhesion molecules on endothelial cells, promotes T cell priming, causes vasodilatation and hypotension (21, 22). Finally, secretion of IL-12 leads to upregulation of the transcription factor T-box expressed in T cells (T-bet) promoting T cell differentiation into interferon-γ producing T helper 1 cells (Th1) (23, 24).

In the past decades, dendritic cell activation, toxicological assessment studies, as well as studies investigating pathophysiological pathways of inflammatory skin diseases have been conducted almost exclusively in animal models, mostly in mice. However, when compared to the situation in humans, species-specific differences in the anatomy, immune cell populations, especially in DC subsets, and pathophysiology are tremendous (25). Furthermore, humans and mice have only ~ 30% of skin-associated genes in common (26), which impairs the translation from mouse models to human skin diseases. Admittedly, human *ex vivo* skin explants are a valuable tool for research; however, they are limited by ethical approval, logistics and high donor and anatomic site variation (27–29). Hence, there is an urgent need for alternative predictive human *in vitro* models.

Immune competent skin models reported to date focus mostly on the integration of Langerhans cell surrogates either derived from the human myeloid leukemia-derived cell line Mutz-3 (30–35), originated from cord-blood derived CD34^+^ hematopoietic progenitor (36, 37) or generated from CD14^+^ peripheral blood mononuclear cells (32). Notably, only one full-thickness skin equivalent has been described containing LC and DDC surrogates derived from CD14^+^ monocytes isolated from peripheral blood mononuclear cells aiming at analyzing the impact of ultraviolet (UV) stress on skin immune cells (38). However, no immune competent human full-thickness model with integrated functional dermal dendritic cells for sensitizer or drug analysis has been described so far. This is surprising as several studies have indicated the crucial role of DDC for antigen presentation in the skin. For instance, after sensitizer treatment dermal dendritic cells might migrate and activate T cells faster and outnumber LCs by 10:1 in draining lymph nodes (39, 40). Furthermore, DDCs might be able to induce a stronger T cell proliferation than LCs (39).

Our previously published results showed that the human monocytic cell line THP-1 can be differentiated into immature dendritic cells (iDCs), displaying a sufficient ability and sensitivity to robustly identify classified proficiency chemicals and model sensitizers such as DNCB and NiSO_4_ *in vitro*. Furthermore, as expected from antigen presenting cells, THP-1-originated iDCs have proven to phagocytose membrane components derived from pathogens such as zymosan and, finally, iDCs might be able to induce T cell activation via upregulation of IL-12p40 upon sensitizer treatment (41). Since THP-1-derived iDCs fulfill all required *in vitro* criteria, we aim to demonstrate in this study the functionality of stably integrated iDCs as dermal dendritic cell surrogates within a human full-thickness skin model and their subsequent molecular characterization before and after sensitizer treatment.

## Materials and methods

### Generation of immature dendritic cells

Immature dendritic cells were generated according to the protocol described by Hölken and Teusch (41). In total, 1 x 10^6^ THP-1 cells were seeded in 5 mL RPMI-1640 (Gibco, #22400089) containing 10% FBS (Gibco, #10270-106), 50 U/mL Pen-Strep (Gibco, #15140122) and 50 µM 2-mercaptoethanol (Gibco, #21985023) into a T25 flask. For the differentiation 1500 IU/mL rhGM-CSF (ImmunoTools, #11343125) and 1500 IU/ml rhIL-4 (ImmunoTools, #11340045) were added with a medium exchange on day 3. The cells were incubated in total for 5 days at 37°C, 5% CO_2_.

### Sensitization assays

1 x 10^6^ THP-1-derived iDCs were seeded into a 24-well plate in 1 mL RPMI-1640 containing 10% FBS, 50 U/ml Pen-Strep, and 50 µM 2-mercaptoethanol. Cells were pre-treated with 1 µM dexamethasone (Peprotech, #5000222) for 1 h. Afterwards cells were treated with 1-chloro-2,4-dinitrobenzene (DNCB) [20 µM] (Sigma-Aldrich, #237329, Darmstadt, Germany), nickel sulfate (NiSO_4_) [380 µM] (Sigma-Aldrich, #227676) or their respective solvent control dimethyl sulfoxide (DMSO). After 24 h, the cells were harvested for the analysis of surface marker expression via flow cytometry.

### Flow cytometry

The cells were harvested after differentiation, treatment or skin model dissociation and washed in Automacs Running Buffer (Miltenyi, #130-091-221). At least 2 x 10^5^ cells for each antibody panel were transferred to 96-well u-bottom plates. For staining the cells were incubated in Automacs Running Buffer with the following antibodies (1:50): REA Control (S)-VioGreen (Miltenyi, #130-113-444), REA Control (S)-PE (Miltenyi, #130-113-438), REA Control (S)-APC (Miltenyi, #130-113-434); REA Control (S)-PE-Vio770, (Miltenyi, #130-113-440); CD54-APC (Miltenyi, #130-121-342); CD86-APC (Miltenyi, #130-116-161), CD14-VioGreen (Miltenyi, #130-110-525), CD11c-APC (Miltenyi, #130-113-584) for 10 minutes in the dark. For single cells from the dissociates skin models the following antibodies were used (1:50): CD45-VioBright R667 (Miltenyi, ##130-110-779), CD54-PE-Vio770 (Miltenyi, #130-127-992), CD86-PE-Vio770 (Miltenyi, #130-116-265). The cells were washed twice with automacs running buffer. To determine the cell viability, cells were stained with DAPI (Sigma, #D9542). Flow cytometry analysis was performed using the CytoFlex (B5-R3-V5) from Beckman Coulter.

### Western blot analysis

THP-1-derived iDCs were seeded with 1 x 10^6^ cells into a 24-well plate in 1 mL RPMI-1640 containing 10% FBS, 50 U/mL Pen-Strep, and 50 µM 2-mercaptoethanol. Cells were treated with DNCB [25 µM] (Sigma-Aldrich, #237329) and NiSO_4_ [500 µM] (Sigma-Aldrich, #227676) for 30 min or 1 h. Cells were harvested, washed once in 1x PBS and lysed with radioimmunoprecipitation assay (RIPA) buffer containing a protease inhibitor (Roche, #11836170001) and a phosphatase inhibitor (Roche, #04906845001). Protein concentration was determined using a BCA protein assay kit (Thermo Scientific, 23227). For western blot analysis, Laemmli buffer (Bio-Rad Laboratories, Inc., #1610747) was added to 20 μg protein lysate. Proteins were denaturated for 10 min at 95°C and separated on 10% SDS-Gels using a Biometra Eco-Mini Buffer Tank system (Analytik Jena, #846-017-103/017-170). Protein transfer to a PVDF membrane (BioRad Laboratories, Inc., ##1620177) was performed with the Biometra Fastblot system (Analytik Jena, #846-015-299). The membrane was blocked in 5% BSA (Roth, #8076.2) and then incubated with the respective primary antibodies, p38 MAPK (Cell Signaling Technology, #8690T), phospho-p38 MAPK (Thr180/Tyr182) (Cell Signaling Technology, #4511T), IκBα (Cell Signaling Technology, #9242S) or vinculin (Cell Signaling Technology, #13901S) over night at 4° C. After washing with 1x TBS-T the membrane was incubated with the respective horseradish peroxidase-coupled secondary antibody (Goat anti-Rabbit (H+L), Thermo Fisher Scientific, #31460) for 1 h at room temperature. Antibody binding was detected with the SuperSignal West Pico Plus substrate kit (Thermo Fisher Scientific, #34577). For imaging we used a ChemStudio Imager (Analytik Jena, #849-97-0928-04).

### qRT-PCR

THP-1-derived iDCs were seeded with 1 x 10^6^ cells into a 24-well plate in 1 mL RPMI-1640 containing 10% FBS, 50 U/mL Pen-Strep, and 50 µM 2-mercaptoethanol. Cells were treated with DNCB [20 µM] (Sigma-Aldrich, #237329) and NiSO_4_ [380 µM] (Sigma-Aldrich, #227676) or their respective solvent control dimethyl sulfoxide (DMSO) for 6 h. RNA-extraction was performed using the RNeasy MiniKit (Qiagen, #74104). Following, the RNA yield and concentration was determined via OD260/280 measurement using the Tecan Spark NanoQuant Plate. For reverse transcription the QuatiTect Reverse Transcription Kit (Qiagen, #205311) was used and a total of 1 µg of RNA was transcribed. Quantitative real-time PCR (qPCR) reactions were performed in triplicates for 50 ng cDNA per sample using Luna Universal qPCR Master Mix (NEB, #M3003L) on a qTower3 G (Analytik Jena, #844-00556-2). The specific primers used were GAPDH (forward, 5′-TGCACCACCAACTGCTTAGC-3′; reverse, 5′-GGCATGGACTGTGGTCATGAG-3′), IL-6 (forward, 5′GGCACTGGCAGAAAACAACC–3′; reverse, 5′-GCAAGTCTCCTCATTGAATCC-3′), IL-8 (forward, 5′-ACTGAGAGTGATTGAGAGTGGAC-3′; reverse, 5′-AACCCTCTGCACCCAGTTTTC-3′), TNF-α (forward, 5′-CCCTGCTGCACTTTGGAGTG-3′; reverse, 5′-TCGGGGTTCGAGAAGATGAT-3′). After amplification, a threshold was set for each gene and Ct values were calculated for all samples.

### Cytokine secretion

THP-1-derived iDCs were seeded with 1 x 10^6^ cells into a 24-well plate in 1 mL RPMI-1640 containing 10% FBS, 50 U/mL Pen-Strep, and 50 µM 2-mercaptoethanol. Cells were treated with DNCB [20 µM] (Sigma-Aldrich, #237329) and NiSO_4_ [380 µM] (Sigma-Aldrich, #227676) or their respective solvent control dimethyl sulfoxide (DMSO). Supernatants were collected after 24 h for cytokine analysis. Secretion of the inflammatory cytokines was detected according to the manufacturer’s instructions of the Cytometric Bead Array Human Inflammatory Cytokines Kit (BD, #551811) and the CytoFlex (B5-R3-V5) from Beckman Coulter. Analysis was performed using the CBA Analysis Software (BD Biosciences).

### 3D immune competent skin model generation

Feeder cells (Phenion, #hFeeder) were seeded with 5 x 10^5^ cells in 23 mL keratinocyte medium (Phenion, #K CM-250) into a T175 flask. After three days 5 x 10^5^ primary human foreskin keratinocytes from juvenile donors (Phenion, #hK P1) were seeded onto the feeder cells. After 6 days of cultivation, feeder cells were detached by incubation with 0.05% trypsin (Gibco, #25300054) for 2 min at 37° C, 5% CO_2_ and keratinocytes were harvested using 0.05% trypsin for 6 min, 37° C, 5% CO_2_. 5 x 10^5^ Keratinocytes in P2 were seeded together with 1 x 10^6^ THP-1-derived iDCs (ratio 1:2) in 1 mL keratinocyte medium (Phenion, #K CM-250) onto dermis models based on a solid and porous collagen matrix (42, 43) and primary human foreskin fibroblasts (kindly provided by Henkel AG & Co. KGaA, Düsseldorf, Germany). After 24 h of incubation at 37° C, 5% CO_2_ the medium was exchanged. After 24 h submerse phase, the skin models were lifted into the Air-liquid Interface and cultivated with Air-liquid Interface Culture Medium (Phenion, #ALI CM HC-250, w/o hydrocortisone) for 10 to 14 days.

### Cryosectioning and immunofluorescence staining

Skin models were embedded and frozen in Tissue-Tek (Sakura, #4583), cut into 7 µm slices and transferred to Microscope slides (expredia, #J1800AMNZ). The tissue slides were fixed in ice-cold acetone for 10 minutes and blocked in 10% normal goat serum (Invitrogen, #50062Z), for 1 h at room temperature. Primary antibodies were diluted in DAKO antibody diluent (Dako, #S0809) and Cytokeratin 5 (OriGene, DM361) (1:75) and CD45-VioBright R667 (Miltenyi, #130-110-779) (1:50) were applied for staining at 4° C overnight. Secondary antibody staining with Alexa Flour 488 (Invitrogen, #A11017) (1:200) combined with DAPI staining (10 µg/ml) (Sigma, ##D9542) was performed for 1 h at room temperature (RT). The stained tissue slides were imbedded with Tissue Fluorescence mounting medium (Agilent, S3023) to avoid bleaching and imaged using confocal spinning disc microscopy (CQ1, Yokogawa). For immunofluorescent staining of the isolated iDCs, cells were transferred into a 96 well plate and fixed with 4% paraformaldehyde (PFA) (Roth, #0964.1) for 10 min. The cells were blocked and permeabilized in 0.1% BSA (Roth, #8076.2), 0.01% Tween20 (Sigma, #P7949) and 0.1% Triton X100 (Sigma, #T9284) in 1x PBS for 30 min. The primary antibody CD45-VioBright R667 was diluted 1:50 in DAKO antibody diluent and applied over night at 4°C. DAPI staining (10 µg/ml) was performed the next day for 1 h at RT. Washing steps were performed each 3x with 1 x PBS at 200 xg for 3 min. Immunofluorescent staining of the cells was analyzed using fluorescence microscopy (Keyence, #BZ-X800L/BZ-X810).

### Hematoxylin and eosin staining

Skin models were fixed in 4% formaldehyde solution (Roth, #P087.5) for at least 24 h before dehydration was conducted in the automated tissue processor (Sakura Finetek USA, Inc., #Tissue-Tek VIP 5 Jr.). For paraffin embedding, samples were processed on Histo Core Arcadia C/H (Leica) and cut into 5 µm sections with the rotary microtome (Leica, #RM2145). Transferred sections on object slides were dried overnight at 37° C in a heating cabinet (Memmert) followed by automated hematoxylin and eosin staining procedure (Thermo Scientific, #Gemini AS). Images were taken with Olympus microscope (BX51, Camera Olympus DP7).

### Skin model dissociation

The immune competent skin models were digested on ALI day 11, 24 h after sensitizer treatment. The tissue was minced via scalpel and tissue scissors and transferred to 1.5 ml tubes. For enzymatic dissociation 1 mL RPMI-1640 containing 10% FBS, 50 U/mL Pen-Strep, 50 µM 2-mercaptoethanol, 100 µg/mL liberase (Roche, #05401119001) and 40 µg/mL DNAse (Roche, #10104159001) was added and the tissue was incubated for 90 min at 37° C, 400 RPM on a thermoshaker (Eppendorf, #PMCT). After 90 minutes, the dissociated cell suspension was filtered through a 70 µm cell strainer (VWR, #732-2758) to obtain single cell suspensions. The cells were washed with PBS and stained for flow cytometry analysis.

### Statistical evaluation

Analysis of the data was conducted with GraphPad Prism version 8.4.3 (GraphPad Software, Inc., San Diego, CA, USA). Statistical significance was calculated using an unpaired t-test, one-way ANOVA or two-way ANOVA. Significance was defined and referred to as * = p ≤ 0.05; ** = p ≤ 0.01; *** = p ≤ 0.001; **** = p ≤ 0.0001.

## Results

**Figure 2:**
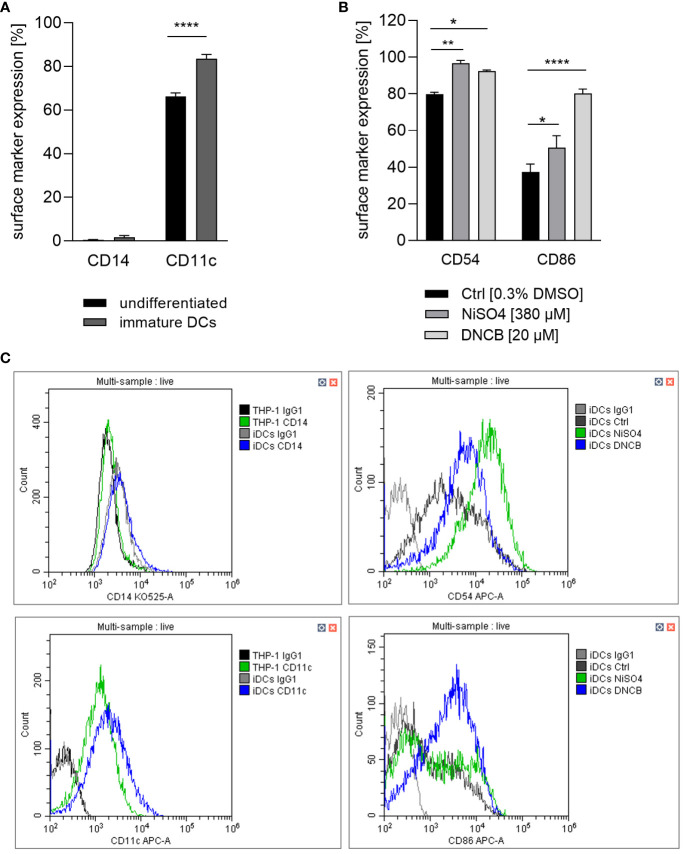
**(A)** CD14 and CD11c surface marker expression of the undifferentiated THP-1 cell line and THP-1-derived iDCs. THP-1 cells were differentiated with 1500 U/ml GM-CSF and 1500 U/ml IL-4 for 5 days, with medium exchange on day 3. **(B)** iDCs were treated with NiSO_4_ [380 µM] and DNCB [20 µM] for 23 h. **(C)** Gating strategy. Surface marker expression (depicted as percentage of positive cells) of at least 10,000 viable cells was analyzed via flow cytometry. Error bars indicate the standard errors of the mean (n = 3 independent experiments with *p ≤ 0.05 and ****p ≤ 0.0001).

When compared to original THP-1 cells our recent study confirmed the expected pronounced ability of THP-1-derived iDCs to phagocytose pathogen membrane particles such as zymosan. Furthermore, iDCs, but not undifferentiated THP-1 cells, are able to induce T cell activation via upregulation of IL-12p40 upon sensitizer treatment (41). Thus, a logical subsequent step is to prove whether the iDCs might be suitable surrogates for dermal dendritic cells. Firstly, the expression of CD11c, which is known to be highly expressed on dermal dendritic cells, and the absence of CD14, a marker for monocytic cells and often used to distinguish DCs from monocytes and macrophages, was investigated. While undifferentiated THP-1 cells, as well as THP-1-derived iDCs express almost no CD14, both express CD11c. However, the expression of CD11c is significantly higher (~1.3-fold) on THP-1-derived iDCs compared to THP-1 cells (**Figures 2A, C**). Hence, our iDCs might be suitable CD14^-^, CD11c^+^ dermal dendritic cell surrogates for the integration into a full-thickness skin model.

**Figure 3:**
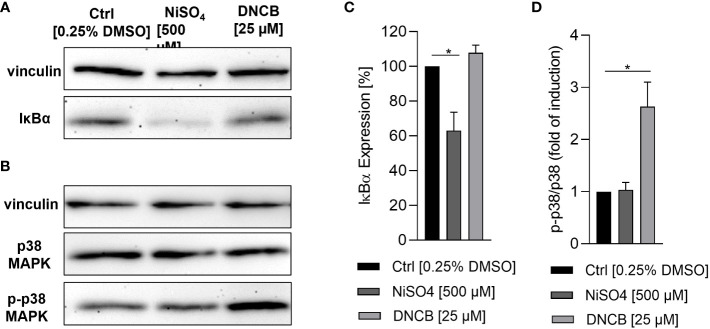
Activation of inflammatory pathways in iDCs after sensitizer treatment. **(A, C)** Degradation of IκBα after NiSO_4_ [500 µM] and DNCB [25 µM] treatment for 1 h, respectively. **(B, D)** Phosphorylation of p38 MAPK after NiSO_4_ [500 µM] and DNCB [25 µM] treatment for 30 min. **(A**, **B)** depict one representative blot of three independent experiments. The housekeeping gene vinculin serves as a loading control. **(C**, **D)** Show the quantification of image bands normalized to the solvent control. Error bars indicate the standard errors of the mean (n = 3 independent experiments with *p ≤ 0.05).

Dendritic cell activation and maturation is characterized by the upregulation of adhesion molecules like CD54 and co-stimulatory molecules and DC maturation markers such as CD86, which are both required for the stimulation of T cells. As expected, treatment of iDCs with NiSO_4_ led to a significantly higher expression of CD54 (~97%) and CD86 (~51%) while DNCB also resulted in a significantly upregulation of CD54 (~93%) and an even higher expression of CD86 (~80%) compared to the solvent control (CD54: ~80%, CD86: ~37%) (**Figures 2B, C**). Furthermore, a few studies demonstrated the activation of the NF-κB pathway in the presence of nickel salts (14, 15). In contrast, for DNCB a phosphorylation of p38 MAPK in DC surrogates was shown (14–16). Moreover, it was proven that both pathways are required for the upregulation of DC activation markers and the subsequent secretion of inflammatory cytokines such as IL-8, IL-6, IL-1 or TNF-α (14–16). We therefore investigated the activation of these pathways as well as the expression of inflammatory cytokines. Treatment of iDCs with 500 µM NiSO_4_ led to significant reduction (1.6-fold) of the inhibitor protein kappa B alpha (IκBα) expression, but (according to previously reported studies) no significant phosphorylation of p38 MAPK could be detected. Contrary, DNCB [25 µM] treatment led to a significant phosphorylation of p38 MAPK (2.6-fold), but no degradation of IκBα (**Figure 3**).

**Figure 4:**
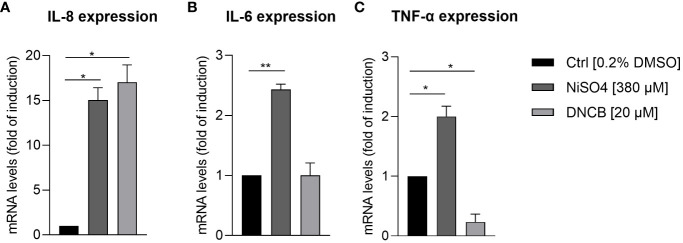
mRNA levels of inflammatory cytokine expression by iDCs: **(A)** IL-8, **(B)** IL-6, **(C)** TNF-α, after NiSO_4_ [380 µM] and DNCB treatment [20 µM] for 6 h. Results are depicted as fold of induction compared to the solvent control [0.2% DMSO] and normalized to the expression of the housekeeping gene [GAPDH]. Error bars indicate the standard errors of the mean (n = 3 independent experiments with *p ≤ 0.05, **p ≤ 0.01).

**Figure 5:**
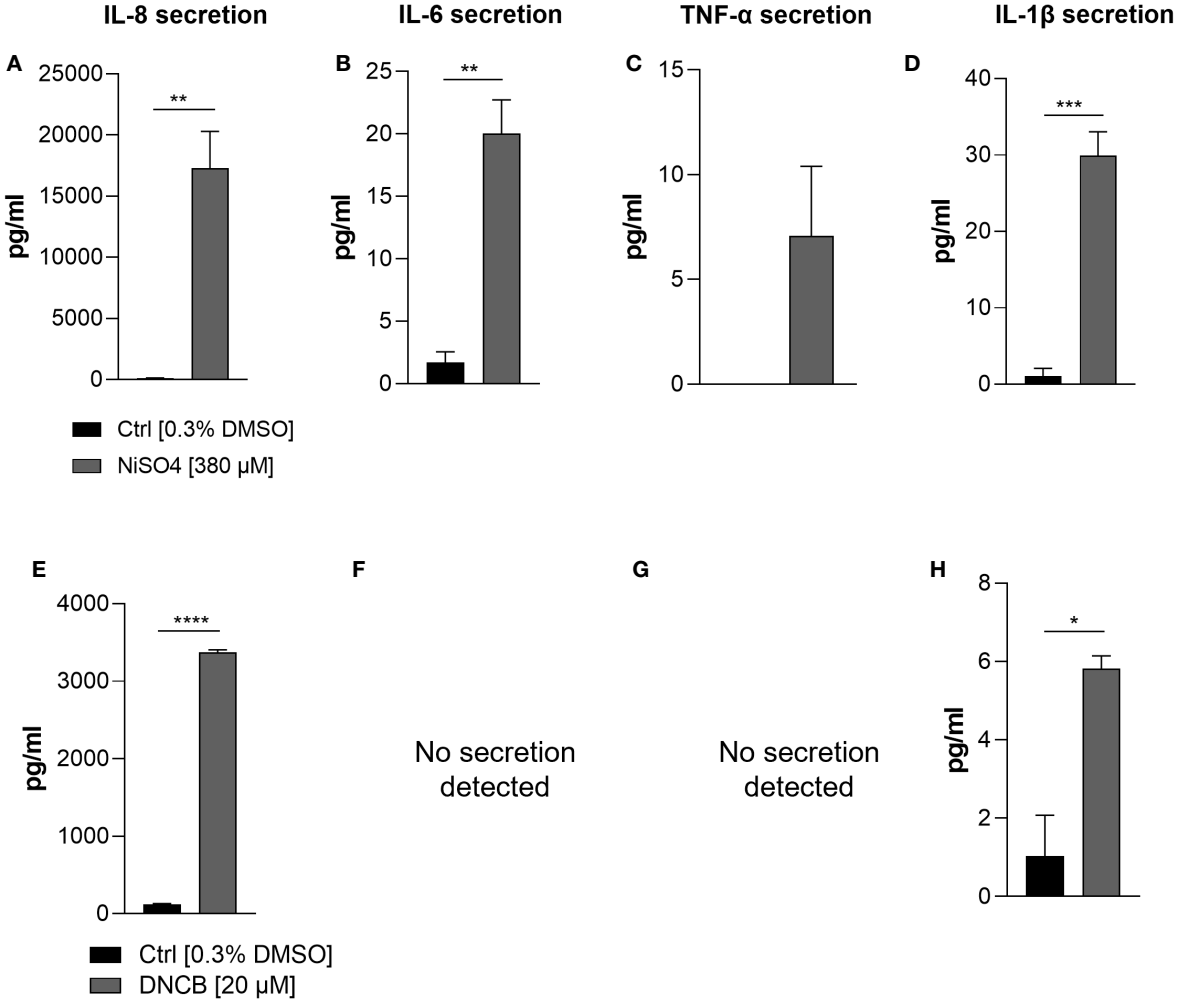
Secretion of inflammatory cytokines: IL-8 **(A, E)**, IL-6 **(B, F)**, TNF-α **(C, G)** IL-1β **(D, H)** by iDCs after NiSO_4_ [380 µM] **(A-D)** and DNCB [20 µM] **(E-H)** treatment. Supernatants were harvested after 24 h and cytokine concentrations were detected using a Cytometric Bead Array Assay. Error bars indicate the standard errors of the mean (n = 3 independent experiments with *p ≤ 0.05, **p ≤ 0.01, ***p ≤ 0.001, and ****p ≤ 0.0001).

In order to prove whether our iDCs are able to up-regulate the expression and to secrete inflammatory cytokines after sensitizer treatment similar to previously reported DC surrogates from various sources, mRNA levels of the inflammatory cytokines IL-8, IL-6 and TNF-α were analyzed. Treatment of iDCs with NiSO_4_ [380 µM] for 6 h induced significant upregulation of mRNA levels for IL-8 (15-fold), IL-6 (2.4-fold) and TNF-α (2-fold). DNCB [20 µM] treatment led to significantly higher IL-8 mRNA levels (17-fold), but no significant upregulation of IL-6 and contrary to NiSO_4_ treatment, significant reduction of TNF-α mRNA levels (0.2-fold) (**Figure 4**). To confirm the mRNA results on the protein level, iDCs were treated for 24 h with 380 µM NiSO_4_ or 20 µM DNCB and the absolute cytokine concentration was determined via a Multiplexing Cytometric Bead Array Assay. Treatment of iDCs with NiSO_4_ induced the secretion of ~17,000 pg/ml IL-8, ~20 pg/ml IL-6, ~30 pg/ml IL-1β and ~7 pg/ml TNF-α, respectively, while DNCB treatment induced the secretion of ~3376 pg/ml IL-8 and ~5.8 pg/ml IL-1β. Secretion of IL-6 and TNF-α could not be detected after DNCB treatment (**Figure 5**). Overall, upon sensitizer treatment THP1-derived iDCs are able to secrete inflammatory cytokines relevant for the activation and recruitment of T cells in the skin, although in different patterns depending on the applied sensitizers.

Next, we aimed to integrate the iDCs into a full-thickness skin model as potential dermal DC surrogates and to prove their functionality *in vitro*. Therefore, iDCs were integrated into the well-established and commercially available Phenion^®^ Full-Thickness Skin Model (44–46). The Phenion^®^ Full-Thickness Skin Model comprises a fully stratified epidermis including a stratum basale, stratum spinosum, stratum granulosum and stratum corneum as well as a mechanically stable dermis. The rigid porous structure allows the fibroblasts to migrate into the scaffold and to synthesize and secrete extracellular matrix components such as elastin and fibrillin-1, mimicking the elastic network of native human skin (44, 47) and thereby potentially providing the organ-specific environment for DDCs.

**Figure 6:**
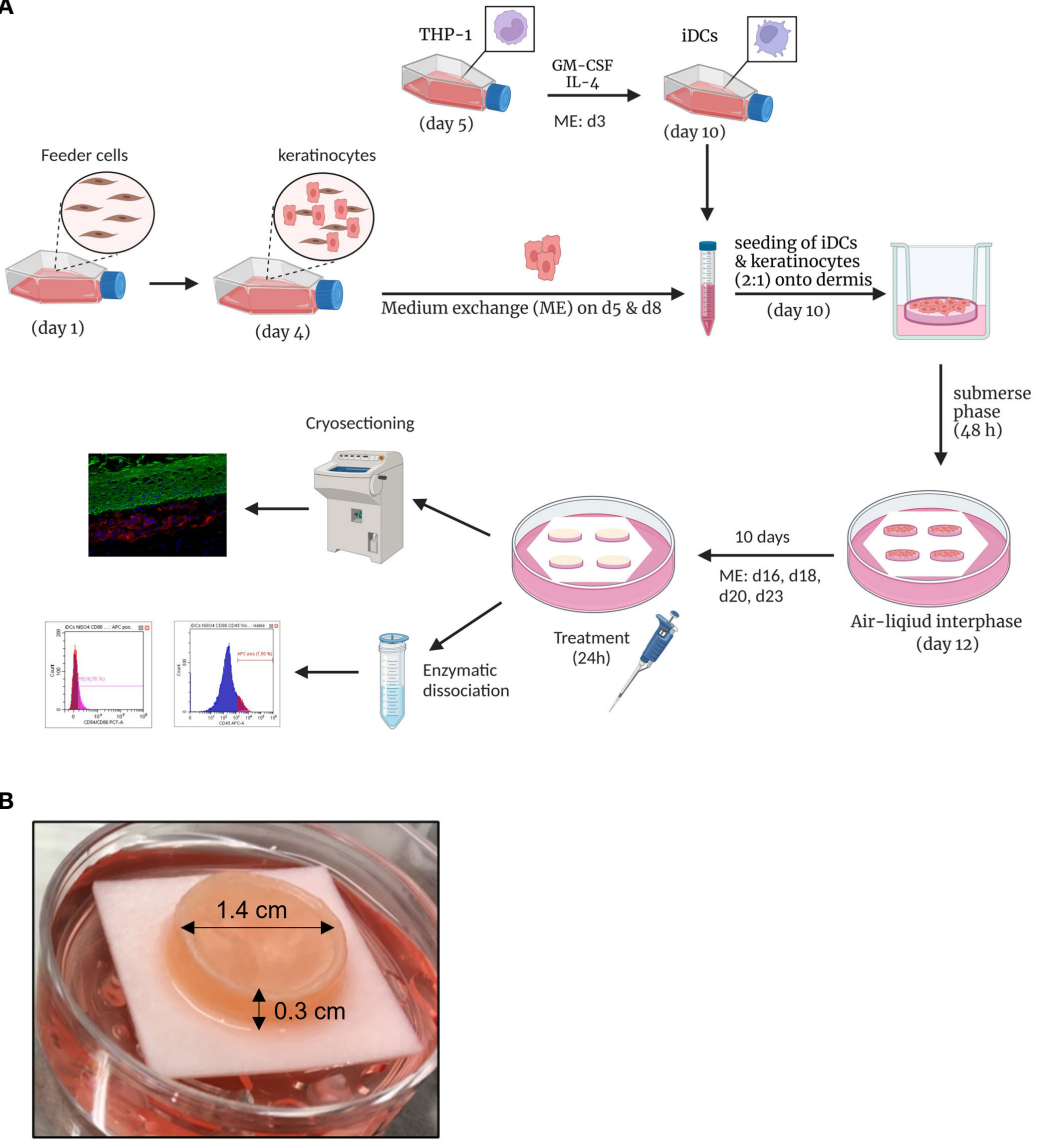
Engineering of human 3D immune competent full-thickness skin models. **(A)** Primary human foreskin keratinocytes are seeded onto feeder cells and harvested after six days of cultivation and seeded together with THP-1-derived iDCs (ratio 1:2) onto dermis models based on a solid collagen matrix and primary fibroblasts. After 48 h of cultivation in a submerse phase, the skin models are lifted into an air-liquid interphase. After 10 days, the skin models are cryo-sectioned for histological analysis or treated with sensitizers for 24 h and enzymatically dissociated. Created with BioRender.com. **(B)** The full-thickness skin model is characterized by a diameter of 1.4 cm and a height of 0.3 cm. The photo was taken on ALI d10 and depicts our immune competent skin model with a fully differentiated epidermis.

**Figure 7:**
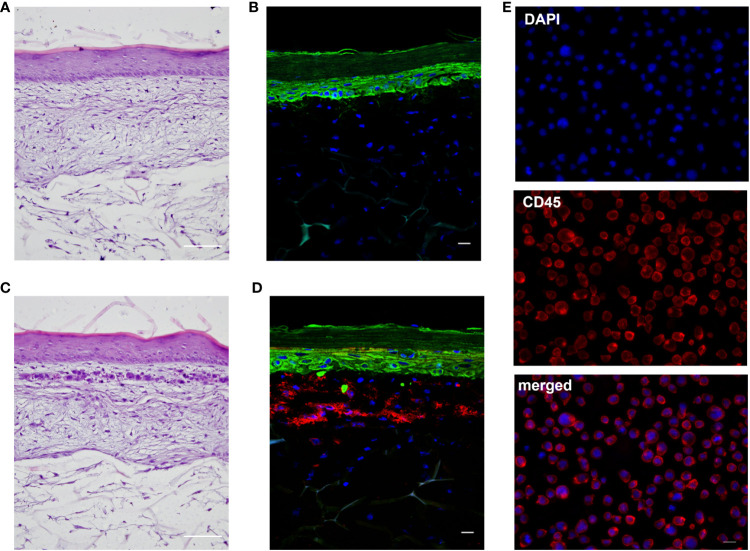
Histological analysis of the full-thickness skin models. **(A**, **C)**: H&E staining of the regular model without immune cells **(A)** and the skin model including DDC surrogates **(C)**. Scale bar = 100 µm. **(B**, **D)**: Immunofluorescent staining of the regular model without immune cells **(B)** and skin model including DDC surrogates **(D)**. Keratinocytes were stained with cytokeratin 5 (green signal). DDC surrogates were stained with CD45 (red signal). Nuclei were stained with DAPI (blue signal). Scale bar = 20 µm. **(E)** Immunofluorescent staining of DDC surrogates before integration into the skin models. DDC surrogates were stained with CD45 (red signal). Nuclei were stained with DAPI (blue signal). Scale bar = 20 µm.

To develop an immune competent skin model, iDCs, were seeded together with primary human keratinocytes onto matured dermis equivalents. After 10 days of air-liquid interphase (ALI) cultivation, allowing the complete differentiation of all epidermal layers, the skin models were either cryo-sectioned for histological analysis or treated with a sensitizer (NiSO_4_ or DNCB) for 24 h and subsequently proceeded towards enzymatic dissociation and DC surface marker analysis (**Figure 6A**). On ALI day 10, the skin model is fully differentiated displaying all epidermal layers typical for native human skin including a stratum corneum, and a dermal compartment enriched with newly synthesized ECM proteins (**Figures 6**, **7A, C**). Histological analysis of the full-thickness skin models reveals the integration of iDCs in the dermis, mostly located underneath the epidermis, which proves the integration as dermal dendritic cells. Compared to the control model, the overall histology of the epidermis is not impaired by the integration of our iDCs and the epidermis remains fully stratified (**Figure 7**).

**Figure 8:**
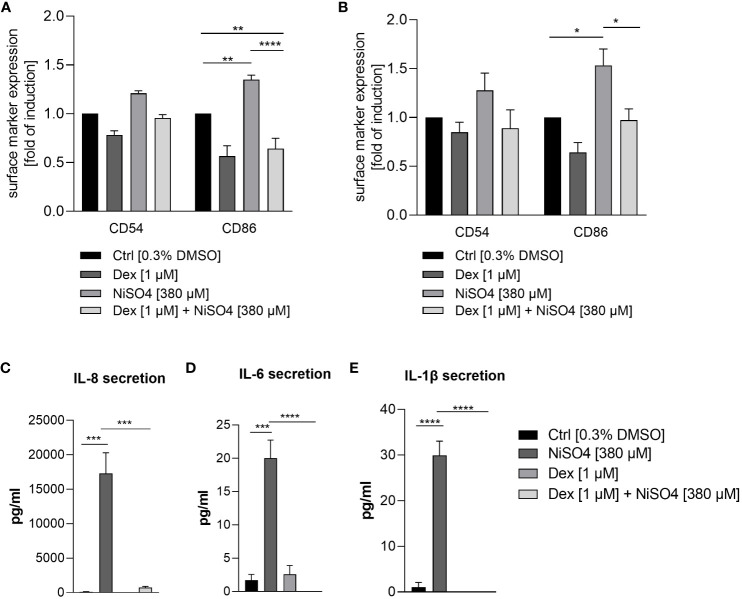
Surface marker expression of CD54 and CD86 (depicted as fold of induction of the percentage of all positive cells) after **(A)** pre-treatment of THP-1-derived iDCs and **(B)** topical treatment of the immune competent skin model with dexamethasone for 1 h, followed by NiSO_4_ treatment for 23 h. Results were depicted as fold of induction compared to the solvent control [0.3% DMSO]. **(C–E)** Cytokine secretion of iDCs after 1 h dexamethasone pre-treatment, followed by 23 h of NiSO_4_ exposure. Error bars indicate the standard errors of the mean (n = 3 independent experiments for **(A, C)** and n=4 independent experiments for **(B)** with *p ≤ 0.05, **p ≤ 0.01, ***p ≤ 0.001, and ****p ≤ 0.0001).

To prove whether iDCs are applicable to a qualitative characterization of sensitizers and perspectively of drug candidates, iDCs were pre-treated for 1 h with dexamethasone, an anti-inflammatory, anti-allergic synthetic glucocorticoid (48), before applying NiSO_4_ [380 µM] for 23 h. Treatment of isolated iDCs with NiSO_4_ alone induced the upregulation of CD54 (~1.2-fold) and a significant upregulation of CD86 (~1.4-fold). The pre-treatment with dexamethasone led to the reduction of the NiSO_4_ induced CD54 (~1.3-fold) expression and a significant reduction of the NiSO_4_ induced CD86 expression (~2.1-fold) (**Figure 8A**) as well as a significant reduction of the IL-8 (23.5-fold), IL-6 (~20-fold) and IL-1β (~30-fold) secretion (**Figure 8C**).

To validate the functionality and immune competence of the DDC surrogate skin model, a topical administration of dexamethasone [1 µM] for 1 h was followed by a topical exposure of NiSO_4_ [380 µM] for 23 h. Afterwards, the skin models were dissociated enzymatically into single cell suspensions and CD45 positive cells were gated for the analysis of the CD54 and CD86 expression on the tissue-integrated iDCs. Topical treatment of the immune competent skin model with NiSO_4_ alone induced a proven upregulation of CD54 (~1.3-fold) and CD86 (~1.5-fold) on the iDCs dissociated from the dermal layer (**Figure 8B**), demonstrating the robust functionality of our dermal DC surrogates *in vitro.* Furthermore, pre-treatment with dexamethasone led to the reduction of the CD54 (~1.4-fold) and a significant reduction of the CD86 (~1.6-fold) expression on iDCs after topical treatment of the immune competent skin model (**Figure 8B**). In conclusion, we were able to engineer a skin model with fully functional dermal dendritic cell surrogates derived from the monocytic cell line THP-1. Furthermore, our immune competent skin model allows the qualitative identification of potential sensitizers and perspectively the evaluation of novel drug candidates potentially suppressing skin sensitization.

## Discussion

The aim of this study was to explore and validate immature dendritic cells (iDCs) derived from the monocytic cell line THP-1 as suitable surrogates for dermal dendritic cells upon integration into a human full-thickness skin model. The ability of THP-1-derived iDCs to identify sensitizers such as NiSO_4_ and DNCB and to upregulate the DC activation markers CD54 and CD86 has been recently shown (41). Furthermore, the capability to phagocytose pathogen-derived membrane components and to potentially induce T cell activation via upregulation of IL-12p40 upon sensitizer treatment has been proven (41). Subsequently, the next logically step was to prove whether our iDCs might be suitable surrogates for dermal dendritic cells.

Dermal dendritic cells can be identified and distinguished from dermal monocytes and macrophages by a low CD14 expression and a high CD11c expression (4, 5). However, in contrast to Langerhans cells, no exclusive cell-specific marker expressed on all dermal dendritic subsets has been reported so far. A commonly described low CD14 expression and a high CD11c expression could be confirmed on the THP-1-derived iDCs. In addition, the significant up-regulation of the DC activation markers CD54 and CD86 after NiSO_4_ and DNCB treatment was verified, confirming the expected ability to respond to sensitizers as a required prerequisite for DC activation and subsequent maturation. Since several studies elucidated the necessity of the activation of the NF-κB pathway and the p38 MAPK pathway for the process of DC activation and maturation marker upregulation (14–16), we studied the impact of the two model sensitizers, NiSO_4_ and DNCB on both pathways in the THP-1-derived iDCs. In line with the published studies, we were able to confirm a significant degradation of IκBα after NiSO_4_ treatment and an induction of phosphorylation of p38 MAPK after DNCB treatment. Treatment of DCs derived from human cord blood with NiSO_4_ led to maximal degradation of IκBα after 1 h and recovery after 4 h, while treatment with DNCB could not induce the degradation of IκBα (14). Similarly, NiCl_2,_ but not DNCB treatment of PBMC-derived DCs for 1 h led to the phosphorylation and degradation of IκBα. In addition, the NiCl_2_-induced activation of NF-κB could be proven via NF-κB p65 transcriptional factor assay kit (15). Conversely, DNCB treatment of human cord blood- derived DCs induced a strong phosphorylation of p38 MAPK after 30 min, while NiSO_4_ treatment could only induce minor phosphorylation of p38 MAPK after 30 min (14). Furthermore, treatment of PBMC derived DCs with DNCB induced a strong dose-dependent phosphorylation of p38 MAPK (15). However, treatment of a fetal mouse skin-derived skin line with NiSO_4_ induced only a weak phosphorylation of p38 MAPK after 2 h of treatment and no degradation of IκBα (49). In fact, sensitization to nickel in mice cannot be achieved without an additional adjuvant to induce the expansion of nickel reactive T cells, while in humans nickel functions as its own adjuvant via the Toll like receptor (TLR) 4, which was identified as receptor for Ni^2+^ in human, but not in mice (50). These results clearly underline the species-specific differences and the necessity to study the skin immunity in human-derived systems. Furthermore, it needs to be mentioned that the TLR4 mediated nickel skin sensitization is most likely guided by dermal dendritic cells, since TLR4 is not expressed on human LCs (51, 52).

Next to the sensitizer induced upregulation of DC activation and maturation markers such as CD54 and CD86 and the activation of the NF-κB as well as the p38 MAPK pathways, the up-regulation and secretion of inflammatory cytokines such as IL-8, IL-6, IL-1 and TNF-α has been described for various DC surrogates (14–16, 53). Thus, we were intrigued to prove the sensitizer- induced expression and secretion of those interleukins in our iDCs as well. Treatment of iDCs with NiSO_4_ resulted in a significant upregulation of mRNA levels for IL-8, IL-6 and TNF-α. These results were confirmed on the protein level, by significant higher cytokine secretion for IL-8, IL-6 and additionally IL-1β. The secretion of IL-8 and IL-6 after NiSO_4_ treatment has been shown for cord blood- derived iDCs as well (14). Furthermore, enhanced mRNA levels as well as cytokine secretion of IL-1β, IL-6, IL-8, TNF-α could be detected after treatment of PBMC derived DCs with NiCl_2_ (15, 53). Treatment of iDCs with DNCB led to a significant upregulation of mRNA levels for IL-8 and IL-6 and significant cytokine secretion for IL-8 and IL-1β. Treatment of PBMC derived iDCs with DNCB resulted in enhanced mRNA levels for IL-8, IL-1β and TNF-α, but only in a significant increased secretion of IL-8 (15). However, treatment of PBMC derived DCs from a different study could prove in line with our results the DNCB induced secretion of IL-1β and no secretion of IL-6 and TNF-α (53). Taken together, our results mirror the results published for other DC surrogates in regard of the p38 MAPK pathway, the NF-κB pathway and inflammatory cytokine induction, suggesting distinct activation mechanisms, different targets and signaling pathways for DNCB compared to nickel salts. Investigating those differences, Ade et al. were able to show that the inhibition of NF-κB with BAY 11-7085 suppressed the NiSO_4_ induced increase of CD86 and CD83 and abolished the NiSO_4_ induced secretion of IL-8, IL-6 and IL12-p40 in cord blood derived DCs (14). However, inhibition of p38 MAPK in PBMC derived DCs with PD98059 suppressed the NiCl_2_ induced IL-1β, IL-8, and TNF-α secretion (15). Inhibition of p38 MAPK in PBMC derived iDCs with SB203580 led to suppressed DNCB induced augmentation of CD86 as well as a suppressed secretion of IL-8 (15). Furthermore, it was shown that DNCB treatment inhibits TNF-α induced activation of the NF-κB pathway in cord blood derived DCs (14). One hypothesis for the distinct mechanisms of action for NiSO_4_ and DNCB could underly their lipophilicity. While DNCB as a lipophilic hapten is able to penetrate directly into the DCs, it can be processed endogenously and presented via MHC class I molecules, hydrophilic nickel ions are more likely processed exogenously and presented via the MHC class II molecules (54–56). In order to elucidate the distinct activation of iDCs upon sensitizer treatment, the molecular mechanism of the haptenization, including the (covalent) binding and modification of proteins followed by the individual, sensitizer or hapten specific DC activation, needs to be addressed in future studies. Although the binding capacity of migratory DCs in skin-draining lymph nodes was proven (57), unfortunately, to date, the precise mechanism of the DNCB and NiSO_4_ DC activation has not been fully established. Furthermore, it has been reported that metal ions such as nickel are bound and presented via different ways to CD4^+^ T cells. While classic allergens such as DNCB tend to form covalent bonds with MHC-bound proteins, metal ions can interact via several molecular mechanisms with T cells (58).

By proving low CD14 and high CD11c expression, the activation of the p38 MAPK and the NF-κB pathway as well as the secretion of inflammatory cytokines after sensitizer treatment in addition to their capability to phagocytose pathogen-derived membrane particles, our THP-1-derived iDCs could be identified as potential dermal dendritic cell surrogates.

For compound characterization, a robust and relatively easily accessible human tissue platform would be desirable as an alternative to animal experiments or highly variable and time-consuming transplants from human skin. Hence, the overall aim was to integrate the iDCs into a human skin model. For this the Phenion^®^ Full-Thickness Skin Model was chosen due its unique porous matrix, which allows the fibroblasts to adhere to and migrate into the collagen and to secrete extracellular matrix components such as elastin and fibrillin-1 (44, 47), mimicking the elastic network of native human skin and thereby potentially providing the inevitable environment for DDCs. Histological analysis of the skin tissue revealed the integration iDCs in the dermis, predominantly underneath the epidermis. This location is in line with the observation for CD11c positive dendritic cells in normal human skin, which have been found to be located in the superior dermis (59). Contrary, cells expressing monocyte/macrophage markers such as CD14 or CD163 are largely located in the superior papillary and reticular dermis (59). Noteworthy, the integration of the iDCs as DDCs did not impair the stratification of the epidermis.

To prove the immune competence of the newly developed iDC containing full-thickness skin model, the skin models were treated topically with 380 µM NiSO_4_ and 20 µM DNCB for 23 h and subsequently dissociated enzymatically into single cell suspensions for the analysis of the surface marker expression of CD54 and CD86 on the DDC surrogates (identified via CD45 expression). While treatment of iDCs with NiSO_4_ only resulted in a 1.2-fold up-regulation of CD54 and a 1.4-fold upregulation of CD86, topical sensitizer administration resulted in a 1.3-fold upregulation of CD54 and a 1.5-fold upregulation of CD86 on tissue-integrated iDCs. Thus, we were able to prove the functionality and thereby the immune competence of our DDC surrogate model 12 days after the integration of iDCs. Noteworthy, despite the vigorous enzymatic dissociation, the expression of both surface markers was still detectable on tissue -integrated iDCs and the upregulation could be detected in a similar manner (fold of induction) compared to the isolated iDCs. In fact, this is not self-evidently, as on one hand the protein expression pattern could change due to the complex three-dimensional co-cultivation with keratinocytes and fibroblasts and on the other hand the enzymatic tissue dissociation has been proven to alter or even diminish the cell surface antigen expression on distinct immune cell populations (60–63).

In order to initially assess the potential of our engineered skin tissue for drug discovery applications, we aimed to prove that our immune competent skin model is amenable for the qualitative characterization of putative anti-inflammatory compounds. Therefore, isolated iDCs as well as the immune competent skin model were treated with dexamethasone, an anti-inflammatory, anti-allergic synthetic glucocorticoid (48), for 1 h followed by 23 h of NiSO_4_ exposure. Indeed, pre-treatment with dexamethasone significantly reduced the NiSO_4_ induced secretion of IL-8, IL-6 and IL-1β and could suppress the expression of CD54 and CD86 on isolated as well as on the tissue-integrated iDCs. In line with our results, the expression of CD54 and CD86 on murine bone marrow derived DC surrogates was downregulated by dexamethasone treatment in a dose-dependent manner and the secretion of IL-1β was decreased significantly (64). Furthermore, the presence of dexamethasone during the differentiation of PBMC into DC surrogates decreased the basal expression of CD86 as well as the TNF-α induced upregulation of CD86 and the LPS-induced secretion of TNF-α and IL-1β (65). By suppressing the expression of CD54 and CD86, as well as the secretion of IL-8, IL-6 and IL-1, which are required for the activation, stimulation and recruitment of T cells, dexamethasone might contribute to T cell inhibitory effects and thereby suppressing the immune response.

Altogether, the THP-1 derived iDCs are profoundly characterized by a low CD14 and high CD11c expression, the ability phagocytose membrane components derived from pathogens (41) and to identify sensitizers such as DNCB and NiSO_4_, which is subsequently followed by the upregulation of adhesion molecules, such as CD54 and co-stimulatory molecules such as CD86 required for the co-stimulation of naïve CD4^+^ T cells. In addition, T cell activation might be supported via upregulation of IL-12p40 upon sensitizer treatment (41). Our findings may contribute to the understanding of the crucial role of DDC for antigen presentation in the skin and the potential to migrate and activate T cells faster and outnumber LCs by 10:1 in draining lymph nodes (39, 40). Furthermore, the sensitizer induced activation of the NF-κB and the p38 MAPK pathway and the secretion of inflammatory cytokines such as IL-8, IL-6, IL-1β and TNF-α as it was stated for other DC surrogates could be validated. Thus, our THP-1-derived iDCs fulfill all required *in vitro* criteria for dermal dendric cell surrogates. By integrating those iDCs into a full-thickness skin model, we are the first to engineer a human immune competent full-thickness skin model containing THP-1-derived iDCs as dermal dendritic cell surrogates, which serve as an easily accessible tool to identify sensitizers and to qualitatively analyze putative anti-inflammatory compounds according to the 3R principles. Prospectively, our immune competent DDC model might be suitable for the research and understanding of inflammatory skin conditions such as psoriasis or diabetic skin manifestations often accompanied with recurring fungal or bacterial infections (66, 67).

## Data availability statement

The raw data supporting the conclusions of this article will be made available by the authors, without undue reservation.

## Ethics statement

Ethical approval was not required for the studies on humans in accordance with the local legislation and institutional requirements because only commercially available established cell lines were used.

## Author contributions

NT: Conceptualization, Funding acquisition, Project administration, Resources, Supervision, Writing – review & editing. JH: Data curation, Investigation, Methodology, Visualization, Writing – original draft. KF: Data curation, Investigation, Validation, Visualization, Writing – review & editing. MM: Validation, Data curation, Investigation, Visualization, Writing – review & editing. NB: Investigation, Data curation, Methodology, Validation, Visualization, Writing – review & editing. UE: Methodology, Validation, Investigation, Visualization, Writing – review & editing. TB: Conceptualization, Funding acquisition, Supervision, Project administration, Resources, Writing – review & editing. KM: Funding acquisition, Methodology, Project administration, Supervision, Resources, Investigation, Writing – review & editing. LV: Data curation, Methodology, Resources, Supervision, Validation, Investigation, Visualization, Writing – review & editing.
